# 4-(3-Chloro­phen­yl)-3-[(2,6-difluoro­benz­yl)sulfan­yl]-5-(3,4,5-trimeth­oxy­phen­yl)-4*H*-1,2,4-triazole

**DOI:** 10.1107/S1600536811048653

**Published:** 2011-11-25

**Authors:** Hoong-Kun Fun, Safra Izuani Jama Asik, B. Chandrakantha, Arun M. Isloor, Prakash Shetty

**Affiliations:** a X-ray Crystallography Unit, School of Physics, Universiti Sains Malaysia, 11800 USM, Penang, Malaysia; b Department of Chemistry, Manipal Institute of Technology, Manipal 576 104, India; c Medicinal Chemistry Division, Department of Chemistry, National Institute of Technology-Karnataka, Surathkal, Mangalore, 575 025, India; d Department of Printing, Manipal Institute of Technology, Manipal 576 104, India

## Abstract

In the title compound, C_24_H_20_ClF_2_N_3_O_3_S, the essentially planar triazole ring (r.m.s. deviation = 0.001 Å) forms dihedral angles of 22.35 (10), 68.17 (10) and 42.01 (10)° with the mean planes of the trimeth­oxy­phenyl, chloro­phenyl and difluoro­phenyl rings, respectively. A weak intra­molecular C—H⋯π inter­action occurs. In the crystal, mol­ecules are linked into sheets lying parallel to the *bc* plane by C—H⋯O and C—H⋯N hydrogen bonds. The crystal packing also features weak C—H⋯π inter­actions.

## Related literature

For the pharmacological activity of [1,2,4] triazole derivatives, see: Zhou *et al.* (2007 ▶); Chen *et al.* (2007 ▶); Isloor *et al.* (2010 ▶); Kalluraya *et al.* (2004 ▶); Sunil *et al.* (2009 ▶); Chandrakantha *et al.* (2010 ▶). For stability of the temperature controller used in the data collection, see: Cosier & Glazer (1986 ▶).
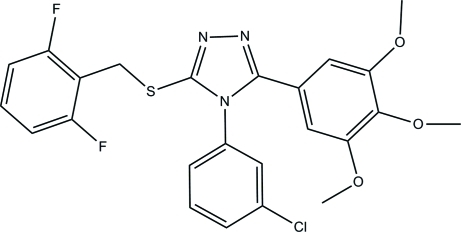


## Experimental

### 

#### Crystal data

C_24_H_20_ClF_2_N_3_O_3_S
*M* *_r_* = 503.94
Monoclinic, 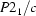

*a* = 9.9867 (2) Å
*b* = 21.5140 (3) Å
*c* = 11.9793 (2) Å
β = 117.197 (1)°
*V* = 2289.24 (7) Å^3^
*Z* = 4
Mo *K*α radiation
μ = 0.31 mm^−1^
*T* = 100 K
0.36 × 0.17 × 0.11 mm

#### Data collection

Bruker SMART APEXII CCD diffractometer
Absorption correction: multi-scan (*SADABS*; Bruker, 2009 ▶) *T*_min_ = 0.896, *T*_max_ = 0.968
26235 measured reflections
6681 independent reflections
5130 reflections with *I* > 2σ(*I*)
*R*_int_ = 0.039

#### Refinement

*R*[*F* ^2^ > 2σ(*F* ^2^)] = 0.049
*wR*(*F* ^2^) = 0.110
*S* = 1.03
6681 reflections
310 parameters
H-atom parameters constrained
Δρ_max_ = 0.43 e Å^−3^
Δρ_min_ = −0.33 e Å^−3^

### 

Data collection: *APEX2* (Bruker, 2009 ▶); cell refinement: *SAINT* (Bruker, 2009 ▶); data reduction: *SAINT*; program(s) used to solve structure: *SHELXTL* (Sheldrick, 2008 ▶); program(s) used to refine structure: *SHELXTL*; molecular graphics: *SHELXTL*; software used to prepare material for publication: *SHELXTL* and *PLATON* (Spek, 2009 ▶).

## Supplementary Material

Crystal structure: contains datablock(s) global, I. DOI: 10.1107/S1600536811048653/hb6503sup1.cif

Structure factors: contains datablock(s) I. DOI: 10.1107/S1600536811048653/hb6503Isup2.hkl

Supplementary material file. DOI: 10.1107/S1600536811048653/hb6503Isup3.cml

Additional supplementary materials:  crystallographic information; 3D view; checkCIF report

## Figures and Tables

**Table 1:** Hydrogen-bond geometry (Å, °) *Cg*2 and *Cg*3 are the centroids of the C1–C6 and C9–C14 rings, respectively.

*D*—H⋯*A*	*D*—H	H⋯*A*	*D*⋯*A*	*D*—H⋯*A*
C11—H11*A*⋯O2^i^	0.95	2.43	3.353 (2)	165
C15—H15*B*⋯O3^ii^	0.99	2.54	3.281 (2)	132
C24—H24*A*⋯N2^iii^	0.98	2.49	3.189 (2)	128
C20—H20*A*⋯*Cg*2^iv^	0.95	2.66	3.543 (2)	154
C1—H1*A*⋯*Cg*3	0.95	2.85	3.6138 (19)	138

Symmetry codes: (i) 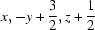; (ii) 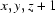; (iii) 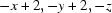; (iv) 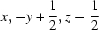.

**Fig. 1.**
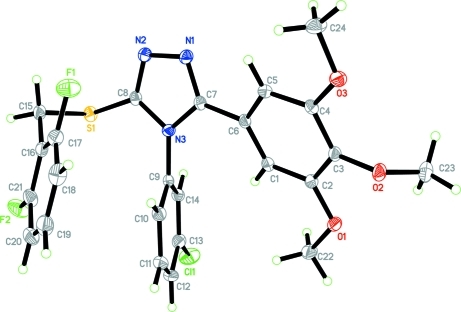
The structure of the title compound, showing 50% probability displacement ellipsoids. Hydrogen atoms are shown as spheres of arbitrary radius.

**Fig. 2.**
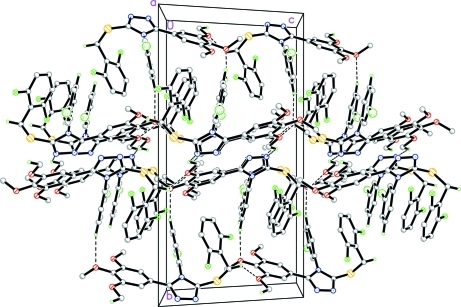
The crystal packing, viewed along the a-axis, showing two-dimensional planes parallel to bc plane. Hydrogen atoms that not involved in hydrogen bonding (dashed lines) are omitted for clarity.

## supplementary crystallographic information

### Comment

During the last few decades, a considerable attention has been devoted to the synthesis of [1, 2, 4] triazole derivatives possessing such diverse pharmacological properties as antimicrobial, anti-inflammatory (Zhou *et al.*, 2007), analgesic antitumorial, antihypertensive (Chen *et al.*, 2007), anticonvulsant and antiviral activities (Isloor *et al.*, 2010). Some 1, 2, 4-triazoles are used as DNA cleaving agents and potassium channel activators. Introduction of fluorine atom in these compounds could alter the course of the pharmacological activities (Kalluraya *et al.*, 2004). In particular, introduction of diflurophenyl substituted group in the moiety immensely increases the pharmacological as well liphophilicity effectiveness (Sunil *et al.*, 2009). It is also observed that the amino and mercapto groups in triazoles are readily accessible nucleophilic centers (Chandrakantha *et al.*, 2010).

In the title compound of (I), (Fig. 1), the triazole (N1–N3/C7/C8) ring is essentially planar, with maximum deviation of 0.001 Å for C1 and N2. The dihedral angles between triazole ring and the mean plane of trimethoxyphenyl (C1–C6/C22–C24/O1–O3), chlorophenyl (C9–C14/Cl1), and difluorophenyl groups (C16–C21/F1–F2) are 22.35 (10), 68.17 (10) and 42.01 (10)° respectively.

In the crystal structure of (Fig. 2), the molecules are linked into two-dimensional network parallel to *bc* plane by C11—H11A···O2, C15—H15B···O3 and C24—H24A···N2 hydrogen bonds. The crystal packing is further stabilized by weak C—H···π interactions (Table 1) with distances of 3.543 (2) and 3.6138 (19) A.

### Experimental

To a solution of 4-(3-chloro phenyl)-5-(3,4,5-trimethoxy phenyl) -4*H*-1,2,4-triazole-3-thiol (1 g, 0.0026 mol) in dry acetonitrile (20 ml) was added potassium carbonate (0.73 g, 0.0053 mol) followed by 2,6-difluorobenzyl bromide (0.58 g, 0.0029 mol) at RT. After the addition, the reaction mixture was stirred at RT for 6h. Reaction mixture was monitored by TLC. After the completion, the reaction mixture was concentrated and purified by column chromatography using pet ether, ethyl acetate as an eluent to afford title compound as colorless solid. Yield: 1.1 g, 84%. M.p. 450-453 K.

### Refinement

All the H atoms were positioned geometrically and refined using a riding model with C–H = 0.93–0.99 Å. The *U*_iso_ values were constrained to be 1.5*U*_eq_ of the carrier atom for methyl H atoms and 1.2*U*_eq_ for the remaining H atoms. A rotating group model was used for the methyl groups.

### Crystal data

**Table:**

C_24_H_20_ClF_2_N_3_O_3_S	*F*(000) = 1040
*M**_r_* = 503.94	*D*_x_ = 1.462 Mg m^−^^3^
Monoclinic, *P*2_1_/*c*	Mo *K*α radiation, λ = 0.71073 Å
Hall symbol: -P 2ybc	Cell parameters from 7706 reflections
*a* = 9.9867 (2) Å	θ = 2.3–30.0°
*b* = 21.5140 (3) Å	µ = 0.31 mm^−^^1^
*c* = 11.9793 (2) Å	*T* = 100 K
β = 117.197 (1)°	Block, colourless
*V* = 2289.24 (7) Å^3^	0.36 × 0.17 × 0.11 mm
*Z* = 4	

### Data collection

**Table:**

Bruker SMART APEXII CCD diffractometer	6681 independent reflections
Radiation source: fine-focus sealed tube	5130 reflections with *I* > 2σ(*I*)
graphite	*R*_int_ = 0.039
φ and ω scans	θ_max_ = 30.0°, θ_min_ = 1.9°
Absorption correction: multi-scan (*SADABS*; Bruker, 2009)	*h* = −13→14
*T*_min_ = 0.896, *T*_max_ = 0.968	*k* = −30→28
26235 measured reflections	*l* = −16→13

### Refinement

**Table:**

Refinement on *F*^2^	Primary atom site location: structure-invariant direct methods
Least-squares matrix: full	Secondary atom site location: difference Fourier map
*R*[*F*^2^ > 2σ(*F*^2^)] = 0.049	Hydrogen site location: inferred from neighbouring sites
*wR*(*F*^2^) = 0.110	H-atom parameters constrained
*S* = 1.03	*w* = 1/[σ^2^(*F*_o_^2^) + (0.0429*P*)^2^ + 1.4878*P*] where *P* = (*F*_o_^2^ + 2*F*_c_^2^)/3
6681 reflections	(Δ/σ)_max_ < 0.001
310 parameters	Δρ_max_ = 0.43 e Å^−^^3^
0 restraints	Δρ_min_ = −0.33 e Å^−^^3^

### Special details

**Table:**

Experimental. The crystal was placed in the cold stream of an Oxford Cryosystems Cobra open-flow nitrogen cryostat (Cosier & Glazer, 1986) operating at 100.0 (1) K.
Geometry. All esds (except the esd in the dihedral angle between two l.s. planes) are estimated using the full covariance matrix. The cell esds are taken into account individually in the estimation of esds in distances, angles and torsion angles; correlations between esds in cell parameters are only used when they are defined by crystal symmetry. An approximate (isotropic) treatment of cell esds is used for estimating esds involving l.s. planes.
Refinement. Refinement of F^2^ against ALL reflections. The weighted R-factor wR and goodness of fit S are based on F^2^, conventional R-factors R are based on F, with F set to zero for negative F^2^. The threshold expression of F^2^ > 2sigma(F^2^) is used only for calculating R-factors(gt) etc. and is not relevant to the choice of reflections for refinement. R-factors based on F^2^ are statistically about twice as large as those based on F, and R- factors based on ALL data will be even larger.

### Fractional atomic coordinates and isotropic or equivalent isotropic displacement parameters (Å^2^)

**Table:**

	*x*	*y*	*z*	*U*_iso_*/*U*_eq_	
Cl1	0.09590 (5)	0.86248 (3)	0.08433 (5)	0.03151 (13)	
S1	0.68053 (5)	0.94142 (2)	0.38418 (4)	0.01701 (10)	
F1	1.00621 (13)	0.87358 (6)	0.32771 (11)	0.0301 (3)	
F2	0.68850 (14)	0.78856 (5)	0.47897 (11)	0.0313 (3)	
O1	0.29452 (13)	0.86279 (6)	−0.34883 (11)	0.0175 (3)	
O2	0.48997 (14)	0.88263 (6)	−0.44702 (11)	0.0175 (3)	
O3	0.75145 (14)	0.93893 (6)	−0.31485 (11)	0.0182 (3)	
N1	0.77291 (16)	0.99061 (7)	0.11532 (13)	0.0162 (3)	
N2	0.79349 (16)	0.99452 (7)	0.23776 (13)	0.0163 (3)	
N3	0.61625 (16)	0.92492 (6)	0.13604 (13)	0.0137 (3)	
C1	0.47268 (19)	0.90647 (8)	−0.14903 (15)	0.0147 (3)	
H1A	0.4059	0.8999	−0.1136	0.018*	
C2	0.42908 (18)	0.89043 (8)	−0.27358 (15)	0.0146 (3)	
C3	0.52434 (19)	0.90190 (8)	−0.32781 (15)	0.0139 (3)	
C4	0.66518 (19)	0.92956 (8)	−0.25484 (15)	0.0143 (3)	
C5	0.71188 (18)	0.94367 (8)	−0.12895 (15)	0.0146 (3)	
H5A	0.8088	0.9609	−0.0791	0.018*	
C6	0.61431 (19)	0.93212 (8)	−0.07673 (15)	0.0136 (3)	
C7	0.66734 (18)	0.94915 (8)	0.05602 (15)	0.0137 (3)	
C8	0.69995 (18)	0.95528 (8)	0.24881 (15)	0.0143 (3)	
C9	0.50775 (19)	0.87636 (8)	0.11320 (14)	0.0139 (3)	
C10	0.5402 (2)	0.81642 (8)	0.09147 (16)	0.0180 (3)	
H10A	0.6346	0.8069	0.0944	0.022*	
C11	0.4324 (2)	0.77023 (9)	0.06527 (16)	0.0216 (4)	
H11A	0.4528	0.7289	0.0494	0.026*	
C12	0.2952 (2)	0.78418 (9)	0.06222 (17)	0.0223 (4)	
H12A	0.2215	0.7527	0.0442	0.027*	
C13	0.2666 (2)	0.84463 (9)	0.08571 (16)	0.0196 (4)	
C14	0.37204 (19)	0.89165 (8)	0.11156 (15)	0.0156 (3)	
H14A	0.3518	0.9329	0.1276	0.019*	
C15	0.8492 (2)	0.89379 (8)	0.47096 (16)	0.0180 (4)	
H15A	0.9389	0.9180	0.4825	0.022*	
H15B	0.8586	0.8847	0.5553	0.022*	
C16	0.84819 (19)	0.83360 (8)	0.40752 (15)	0.0174 (3)	
C17	0.9269 (2)	0.82486 (9)	0.33895 (16)	0.0201 (4)	
C18	0.9289 (2)	0.76939 (9)	0.28106 (17)	0.0251 (4)	
H18A	0.9852	0.7655	0.2354	0.030*	
C19	0.8471 (2)	0.71991 (9)	0.29131 (18)	0.0270 (4)	
H19A	0.8467	0.6815	0.2521	0.032*	
C20	0.7655 (2)	0.72574 (9)	0.35825 (17)	0.0256 (4)	
H20A	0.7092	0.6918	0.3657	0.031*	
C21	0.7685 (2)	0.78214 (9)	0.41376 (16)	0.0208 (4)	
C22	0.2031 (2)	0.84505 (9)	−0.29081 (17)	0.0204 (4)	
H22A	0.1137	0.8230	−0.3518	0.031*	
H22B	0.1720	0.8823	−0.2616	0.031*	
H22C	0.2609	0.8178	−0.2193	0.031*	
C23	0.3695 (2)	0.91553 (10)	−0.54687 (16)	0.0236 (4)	
H23A	0.3463	0.8955	−0.6272	0.035*	
H23B	0.4001	0.9587	−0.5485	0.035*	
H23C	0.2801	0.9148	−0.5331	0.035*	
C24	0.8921 (2)	0.97061 (9)	−0.24538 (18)	0.0237 (4)	
H24A	0.9396	0.9779	−0.2999	0.036*	
H24B	0.9586	0.9450	−0.1737	0.036*	
H24C	0.8740	1.0105	−0.2151	0.036*	

### Atomic displacement parameters (Å^2^)

**Table:**

	*U*^11^	*U*^22^	*U*^33^	*U*^12^	*U*^13^	*U*^23^
Cl1	0.0222 (2)	0.0397 (3)	0.0385 (3)	−0.0091 (2)	0.0190 (2)	−0.0078 (2)
S1	0.0213 (2)	0.0192 (2)	0.01326 (19)	0.00148 (17)	0.01018 (16)	−0.00077 (16)
F1	0.0286 (6)	0.0313 (7)	0.0378 (7)	−0.0063 (5)	0.0217 (5)	−0.0054 (5)
F2	0.0480 (8)	0.0257 (6)	0.0333 (6)	−0.0054 (5)	0.0299 (6)	−0.0001 (5)
O1	0.0166 (6)	0.0213 (7)	0.0138 (6)	−0.0053 (5)	0.0063 (5)	−0.0016 (5)
O2	0.0221 (6)	0.0185 (6)	0.0120 (5)	0.0011 (5)	0.0080 (5)	−0.0016 (5)
O3	0.0198 (6)	0.0215 (6)	0.0170 (6)	−0.0054 (5)	0.0115 (5)	−0.0030 (5)
N1	0.0173 (7)	0.0187 (8)	0.0126 (7)	−0.0025 (6)	0.0068 (6)	−0.0017 (5)
N2	0.0183 (7)	0.0172 (7)	0.0131 (6)	−0.0016 (6)	0.0070 (6)	−0.0012 (5)
N3	0.0149 (6)	0.0134 (7)	0.0129 (6)	−0.0018 (5)	0.0064 (5)	−0.0013 (5)
C1	0.0160 (8)	0.0144 (8)	0.0136 (7)	0.0010 (6)	0.0066 (6)	0.0020 (6)
C2	0.0147 (8)	0.0132 (8)	0.0139 (8)	0.0007 (6)	0.0048 (6)	0.0015 (6)
C3	0.0176 (8)	0.0125 (8)	0.0109 (7)	0.0010 (6)	0.0060 (6)	−0.0002 (6)
C4	0.0164 (8)	0.0145 (8)	0.0143 (8)	−0.0001 (6)	0.0091 (6)	0.0016 (6)
C5	0.0152 (8)	0.0130 (8)	0.0148 (8)	−0.0012 (6)	0.0061 (6)	−0.0001 (6)
C6	0.0159 (7)	0.0120 (8)	0.0121 (7)	0.0013 (6)	0.0057 (6)	0.0015 (6)
C7	0.0153 (7)	0.0146 (8)	0.0122 (7)	0.0012 (6)	0.0070 (6)	0.0010 (6)
C8	0.0165 (8)	0.0146 (8)	0.0120 (7)	0.0018 (6)	0.0068 (6)	−0.0004 (6)
C9	0.0169 (8)	0.0148 (8)	0.0092 (7)	−0.0024 (6)	0.0053 (6)	0.0012 (6)
C10	0.0200 (8)	0.0157 (8)	0.0161 (8)	0.0013 (7)	0.0066 (7)	0.0005 (7)
C11	0.0283 (10)	0.0138 (8)	0.0188 (8)	−0.0022 (7)	0.0074 (7)	0.0004 (7)
C12	0.0261 (9)	0.0193 (9)	0.0190 (9)	−0.0098 (7)	0.0082 (7)	0.0005 (7)
C13	0.0194 (8)	0.0248 (10)	0.0157 (8)	−0.0039 (7)	0.0091 (7)	0.0001 (7)
C14	0.0189 (8)	0.0155 (8)	0.0135 (8)	−0.0013 (6)	0.0084 (6)	−0.0003 (6)
C15	0.0219 (9)	0.0170 (9)	0.0122 (8)	0.0002 (7)	0.0052 (7)	−0.0004 (6)
C16	0.0190 (8)	0.0162 (8)	0.0125 (8)	0.0018 (7)	0.0034 (6)	0.0006 (6)
C17	0.0185 (8)	0.0215 (9)	0.0184 (8)	−0.0004 (7)	0.0068 (7)	0.0001 (7)
C18	0.0259 (10)	0.0288 (11)	0.0206 (9)	0.0078 (8)	0.0105 (8)	−0.0015 (8)
C19	0.0343 (11)	0.0197 (10)	0.0210 (9)	0.0074 (8)	0.0075 (8)	−0.0015 (7)
C20	0.0332 (11)	0.0176 (9)	0.0217 (9)	0.0011 (8)	0.0087 (8)	0.0032 (7)
C21	0.0285 (10)	0.0198 (9)	0.0160 (8)	0.0023 (7)	0.0118 (7)	0.0030 (7)
C22	0.0168 (8)	0.0252 (10)	0.0196 (9)	−0.0050 (7)	0.0087 (7)	−0.0011 (7)
C23	0.0211 (9)	0.0335 (11)	0.0142 (8)	0.0018 (8)	0.0065 (7)	0.0021 (7)
C24	0.0233 (9)	0.0285 (10)	0.0242 (9)	−0.0108 (8)	0.0151 (8)	−0.0085 (8)

### Geometric parameters (Å, °)

**Table:**

Cl1—C13	1.7404 (19)	C10—H10A	0.9500
S1—C8	1.7446 (17)	C11—C12	1.386 (3)
S1—C15	1.8352 (18)	C11—H11A	0.9500
F1—C17	1.357 (2)	C12—C13	1.388 (3)
F2—C21	1.355 (2)	C12—H12A	0.9500
O1—C2	1.366 (2)	C13—C14	1.389 (2)
O1—C22	1.429 (2)	C14—H14A	0.9500
O2—C3	1.3714 (19)	C15—C16	1.499 (2)
O2—C23	1.436 (2)	C15—H15A	0.9900
O3—C4	1.366 (2)	C15—H15B	0.9900
O3—C24	1.436 (2)	C16—C17	1.385 (3)
N1—C7	1.314 (2)	C16—C21	1.386 (3)
N1—N2	1.3875 (19)	C17—C18	1.385 (3)
N2—C8	1.310 (2)	C18—C19	1.381 (3)
N3—C7	1.378 (2)	C18—H18A	0.9500
N3—C8	1.385 (2)	C19—C20	1.385 (3)
N3—C9	1.439 (2)	C19—H19A	0.9500
C1—C6	1.391 (2)	C20—C21	1.377 (3)
C1—C2	1.393 (2)	C20—H20A	0.9500
C1—H1A	0.9500	C22—H22A	0.9800
C2—C3	1.397 (2)	C22—H22B	0.9800
C3—C4	1.405 (2)	C22—H22C	0.9800
C4—C5	1.393 (2)	C23—H23A	0.9800
C5—C6	1.400 (2)	C23—H23B	0.9800
C5—H5A	0.9500	C23—H23C	0.9800
C6—C7	1.474 (2)	C24—H24A	0.9800
C9—C10	1.383 (2)	C24—H24B	0.9800
C9—C14	1.386 (2)	C24—H24C	0.9800
C10—C11	1.392 (3)		
			
C8—S1—C15	99.14 (8)	C14—C13—Cl1	118.74 (14)
C2—O1—C22	116.74 (13)	C9—C14—C13	117.84 (16)
C3—O2—C23	115.73 (13)	C9—C14—H14A	121.1
C4—O3—C24	116.76 (13)	C13—C14—H14A	121.1
C7—N1—N2	107.89 (13)	C16—C15—S1	113.79 (12)
C8—N2—N1	107.45 (13)	C16—C15—H15A	108.8
C7—N3—C8	104.48 (13)	S1—C15—H15A	108.8
C7—N3—C9	128.96 (14)	C16—C15—H15B	108.8
C8—N3—C9	126.46 (14)	S1—C15—H15B	108.8
C6—C1—C2	119.75 (15)	H15A—C15—H15B	107.7
C6—C1—H1A	120.1	C17—C16—C21	114.80 (16)
C2—C1—H1A	120.1	C17—C16—C15	122.94 (16)
O1—C2—C1	123.12 (15)	C21—C16—C15	122.26 (16)
O1—C2—C3	116.34 (14)	F1—C17—C18	118.26 (17)
C1—C2—C3	120.54 (15)	F1—C17—C16	117.97 (16)
O2—C3—C2	122.08 (15)	C18—C17—C16	123.77 (18)
O2—C3—C4	118.62 (15)	C19—C18—C17	118.37 (18)
C2—C3—C4	119.10 (15)	C19—C18—H18A	120.8
O3—C4—C5	123.91 (15)	C17—C18—H18A	120.8
O3—C4—C3	115.34 (14)	C18—C19—C20	120.65 (18)
C5—C4—C3	120.70 (15)	C18—C19—H19A	119.7
C4—C5—C6	119.21 (15)	C20—C19—H19A	119.7
C4—C5—H5A	120.4	C21—C20—C19	118.10 (18)
C6—C5—H5A	120.4	C21—C20—H20A	121.0
C1—C6—C5	120.63 (15)	C19—C20—H20A	121.0
C1—C6—C7	122.13 (15)	F2—C21—C20	118.68 (17)
C5—C6—C7	117.23 (15)	F2—C21—C16	117.01 (16)
N1—C7—N3	109.96 (14)	C20—C21—C16	124.31 (18)
N1—C7—C6	123.75 (15)	O1—C22—H22A	109.5
N3—C7—C6	126.29 (15)	O1—C22—H22B	109.5
N2—C8—N3	110.22 (14)	H22A—C22—H22B	109.5
N2—C8—S1	126.26 (13)	O1—C22—H22C	109.5
N3—C8—S1	123.52 (13)	H22A—C22—H22C	109.5
C10—C9—C14	122.02 (16)	H22B—C22—H22C	109.5
C10—C9—N3	119.47 (15)	O2—C23—H23A	109.5
C14—C9—N3	118.49 (15)	O2—C23—H23B	109.5
C9—C10—C11	118.98 (17)	H23A—C23—H23B	109.5
C9—C10—H10A	120.5	O2—C23—H23C	109.5
C11—C10—H10A	120.5	H23A—C23—H23C	109.5
C12—C11—C10	120.31 (17)	H23B—C23—H23C	109.5
C12—C11—H11A	119.8	O3—C24—H24A	109.5
C10—C11—H11A	119.8	O3—C24—H24B	109.5
C11—C12—C13	119.34 (17)	H24A—C24—H24B	109.5
C11—C12—H12A	120.3	O3—C24—H24C	109.5
C13—C12—H12A	120.3	H24A—C24—H24C	109.5
C12—C13—C14	121.50 (17)	H24B—C24—H24C	109.5
C12—C13—Cl1	119.76 (14)		
			
C7—N1—N2—C8	0.07 (18)	C9—N3—C8—N2	176.61 (15)
C22—O1—C2—C1	−5.8 (2)	C7—N3—C8—S1	−179.61 (12)
C22—O1—C2—C3	173.75 (15)	C9—N3—C8—S1	−3.0 (2)
C6—C1—C2—O1	177.28 (15)	C15—S1—C8—N2	−76.25 (16)
C6—C1—C2—C3	−2.3 (2)	C15—S1—C8—N3	103.35 (15)
C23—O2—C3—C2	71.3 (2)	C7—N3—C9—C10	65.5 (2)
C23—O2—C3—C4	−113.93 (18)	C8—N3—C9—C10	−110.22 (19)
O1—C2—C3—O2	−4.5 (2)	C7—N3—C9—C14	−113.38 (19)
C1—C2—C3—O2	175.11 (15)	C8—N3—C9—C14	70.9 (2)
O1—C2—C3—C4	−179.25 (15)	C14—C9—C10—C11	1.0 (2)
C1—C2—C3—C4	0.3 (2)	N3—C9—C10—C11	−177.84 (15)
C24—O3—C4—C5	−6.1 (2)	C9—C10—C11—C12	−0.6 (3)
C24—O3—C4—C3	176.26 (15)	C10—C11—C12—C13	−0.1 (3)
O2—C3—C4—O3	4.8 (2)	C11—C12—C13—C14	0.4 (3)
C2—C3—C4—O3	179.76 (15)	C11—C12—C13—Cl1	−179.09 (14)
O2—C3—C4—C5	−172.94 (15)	C10—C9—C14—C13	−0.7 (2)
C2—C3—C4—C5	2.0 (2)	N3—C9—C14—C13	178.12 (14)
O3—C4—C5—C6	−179.94 (15)	C12—C13—C14—C9	0.0 (3)
C3—C4—C5—C6	−2.4 (2)	Cl1—C13—C14—C9	179.49 (12)
C2—C1—C6—C5	1.9 (2)	C8—S1—C15—C16	−63.03 (14)
C2—C1—C6—C7	−179.10 (15)	S1—C15—C16—C17	100.76 (18)
C4—C5—C6—C1	0.4 (2)	S1—C15—C16—C21	−79.58 (19)
C4—C5—C6—C7	−178.63 (15)	C21—C16—C17—F1	179.26 (15)
N2—N1—C7—N3	−0.04 (19)	C15—C16—C17—F1	−1.1 (3)
N2—N1—C7—C6	−179.95 (15)	C21—C16—C17—C18	−0.5 (3)
C8—N3—C7—N1	0.00 (18)	C15—C16—C17—C18	179.14 (17)
C9—N3—C7—N1	−176.45 (16)	F1—C17—C18—C19	−179.36 (16)
C8—N3—C7—C6	179.90 (16)	C16—C17—C18—C19	0.4 (3)
C9—N3—C7—C6	3.5 (3)	C17—C18—C19—C20	−0.2 (3)
C1—C6—C7—N1	−156.64 (17)	C18—C19—C20—C21	0.1 (3)
C5—C6—C7—N1	22.4 (2)	C19—C20—C21—F2	179.65 (17)
C1—C6—C7—N3	23.5 (3)	C19—C20—C21—C16	−0.2 (3)
C5—C6—C7—N3	−157.48 (16)	C17—C16—C21—F2	−179.45 (15)
N1—N2—C8—N3	−0.08 (19)	C15—C16—C21—F2	0.9 (3)
N1—N2—C8—S1	179.57 (12)	C17—C16—C21—C20	0.4 (3)
C7—N3—C8—N2	0.05 (18)	C15—C16—C21—C20	−179.24 (17)

### Hydrogen-bond geometry (Å, °)

**Table:**

Cg2 and Cg3 are the centroids of the C1–C6 and C9–C14 rings, respectively.

**Table:**

*D*—H···*A*	*D*—H	H···*A*	*D*···*A*	*D*—H···*A*
C11—H11A···O2^i^	0.95	2.43	3.353 (2)	165
C15—H15B···O3^ii^	0.99	2.54	3.281 (2)	132
C24—H24A···N2^iii^	0.98	2.49	3.189 (2)	128
C20—H20A···Cg2^iv^	0.95	2.66	3.543 (2)	154
C1—H1A···Cg3	0.95	2.85	3.6138 (19)	138

Symmetry codes: (i) *x*, −*y*+3/2, *z*+1/2; (ii) *x*, *y*, *z*+1; (iii) −*x*+2, −*y*+2, −*z*; (iv) *x*, −*y*+1/2, *z*−1/2.
